# Multiomics Reveals the Regulatory Mechanisms of Arabidopsis Tissues under Heat Stress

**DOI:** 10.3390/ijms241311081

**Published:** 2023-07-04

**Authors:** Haolang Chen, Mingxi Guo, Mingyang Cui, Yu Yu, Jie Cui, Chao Liang, Lin Liu, Beixin Mo, Lei Gao

**Affiliations:** Guangdong Provincial Key Laboratory for Plant Epigenetics, Longhua Bioindustry and Innovation Research Institute, College of Life Sciences and Oceanography, Shenzhen University, Shenzhen 518060, China; 2100251008@email.szu.edu.cn (H.C.); guomingxi2020@email.szu.edu.cn (M.G.); 2200251043@email.szu.edu.cn (M.C.); yuy@szu.edu.cn (Y.Y.); cuijie@szu.edu.cn (J.C.); chaoliang@szu.edu.cn (C.L.); linliu@szu.edu.cn (L.L.); bmo@szu.edu.cn (B.M.)

**Keywords:** multiomics, Arabidopsis, heat stress, leaves, seedlings, seed

## Abstract

Understanding the mechanisms of responses to high temperatures in Arabidopsis will provide insights into how plants may mitigate heat stress under global climate change. And exploring the interconnections of different modification levels in heat stress response could help us to understand the molecular mechanism of heat stress response in Arabidopsis more comprehensively and precisely. In this paper, we combined multiomics analyses to explore the common heat stress-responsive genes and specific heat-responsive metabolic pathways in Arabidopsis leaf, seedling, and seed tissues. We found that genes such as *AT1G54050* play a role in promoting proper protein folding in response to HS (Heat stress). In addition, it was revealed that the binding profile of *A1B* is altered under elevated temperature conditions. Finally, we also show that two microRNAs, ath-mir156h and ath-mir166b-5p, may be core regulatory molecules in HS. Also elucidated that under HS, plants can regulate specific regulatory mechanisms, such as oxygen levels, by altering the degree of CHH methylation.

## 1. Introduction

High temperature poses a huge challenge to the survival of plants. A large number of important factors and protein families are involved in heat stress responses. These molecules and the regulatory mechanisms of heat stress have been extensively studied in plants [1]. Therefore, it is important to understand and further elucidate the molecular and physiological mechanisms of plant adaptations to elevated temperatures [1]. Omics approaches have been utilized to explore the regulatory mechanisms of plant heat stress, including chromatin remodeling, histone and DNA methylation at the DNA level, non-coding RNAs (ncRNAs), HSFs (heat shock transcription factors), and HSPs (heat shock transcription factors) at the RNA level, and hormones, metabolites, and amino acids at the protein level [2,3,4,5,6,7]. The DNA, RNA, and protein levels of regulation are interrelated, and each plays an important role [1].

For example, microRNAs (miRNAs) have been found to play a key regulatory role in high-temperature stress responses. Among these, the most widely studied are the miR156/miR172, miR159/miR319, miR160/miR393, and miR398 families [2]. Specifically, miR172 plays a role in a temperature-sensing pathway [3]. In addition, HSFs and HSPs are found in most organisms. Exposure to high temperatures stimulates an organism to protect itself from the heat. Many HSPs act as molecular chaperones. The heat stress response of plants involves 21 known transcription factors and 4 HSP families, including HSP110, HSP90, HSP70, and HSP60 [4]. Under normal conditions, HSPs bind to and inactivate HSFs. During heat stress (HS), HSPs dissociate from HSFs and then repair or remove damaged proteins [5,6,7]. The release of HSFs from HSPs allows for polymerization and then binding to heat stress elements (HSEs) in the promoters of HSPs and other target genes. This activation promotes increased production of HSPs to protect cells [8]. In addition, DNA methylation is involved in the response to heat stress. Indeed, exposure to heat stress resulted in increased global methylation and a higher frequency of homologous recombination in plants [9]. In plants, the two main functions of methylation are the protection of the genome from selfish DNA elements and the regulation of gene expression. DNA methylation represses transposon transposition and promotes transcriptional gene silencing (TGs) to maintain genomic stability [10]. In general, DNA methylation acts as a “buffer” under HS, helping to maintain the balance between the expression of genes associated with heat stimulation and the acute activation of transposable elements (TEs) [11]. The activation of heat shock genes and heritable TEs contributes to the establishment and maintenance of plant HS memory and improves the heat tolerance of plants under future HS conditions [1].

Regulatory mechanisms at the RNA level have been investigated primarily by RNA sequencing (RNA-seq). RNA-seq is used most commonly to analyze differentially expressed genes (DEGs) or differentially expressed miRNAs (demiRNAs). Xin et al. compared the expression profiles of miRNA families in tam107 wheat before and after HS [12]. Nine of the 32 miRNA families were identified as thermo-responsive. For example, mir172 significantly decreased by 1.5-fold, while eight miRNAs, including mir156 and mir159, were up-regulated [12]. Barciszewska et al. explored the changes of miRNAs under multiple conditions, such as high-temperature stress, by small RNA sequencing (sRNA-seq) [13]. The miRNAs miR319a/b, miR319b.2, and miR400 were responsive to several abiotic stresses and thus can be regarded as a general type of stress-responsive miRNA [13]. Using RNA-seq, Liu et al. found a subset of genes that were more actively transcribed and had the highest mRNA levels in Arabidopsis leaves after heat treatment [14]. These genes are likely to be the first set of genes responding to HS because they were simultaneously transcriptionally induced by exposure of leaves to 45 °C for 5 min [14]. From the sequencing of the Arabidopsis transcriptome, it was found that heat induced two well-studied inhibitors of protein-coding genes (PCGs) under epigenetic control, SUPPRESSOR OF DRM1 DRM2 CMT3 (SDC) and QUA-QUINE STARCH, which suggests that HS can be associated with epigenetic changes through gene induction [15].

DNA sequencing rapidly identifies a large number of nucleic acid sequences. Modern DNA sequencing technologies allow for the sequencing of the complete genomes of many biological species. For example, bisulfite sequencing (BS-seq) and chromatin immunoprecipitation sequencing (chip-seq) have been used to gain insight into the regulation of DNA through changes in the extent and location of DNA methylation and the exploration of transcription factor binding sites.

The interaction between proteins and DNA can be analyzed with chip-seq [16,17]. Chip-seq combines chromatin immunoprecipitation (chip) with massive parallel DNA sequencing to identify and locate the binding sites of related proteins on the whole genome. High temperatures induced increased small ubiquitin-like modifier (SUMO) conjugation to chromatin regions facilitated by the SUMO ligase SIZ1 in Arabidopsis [18,19]. Using chip-seq, Han et al. showed that SUMO-associated peaks of siz1-2 (a null mutant of SIZ1) were almost absent under normal and HS conditions [20]. This suggests that SIZ1 is required for maintaining the majority of SUMO1/2 occupancy on plant cell chromatin [20]. Cortijo et al. found that the transcriptome of Arabidopsis is dependent on the HSFA1 group of HSFs under HS conditions, which results in the rapid and dynamic removal of H2A.Z nucleosomes at target genes [21]. Transcriptional cascades lead to the activation of multiple downstream stress-responsive transcription factors, triggering large-scale changes in the transcriptome [21].

BS-seq, where DNA is treated with bisulfite followed by in silico sequencing, determines methylation patterns. Studies have shown that ros1 plays a role in later embryonic development by acting on the demethylation of endosperm-specific methylated regions [22,23]. Seed germination is affected by HS, and ros1-4 (a ros1 loss-of-function mutant) produces a large reduction in heat resistance as well as abnormal germination phenotypes under severe stress. Analysis of changes in DNA methylation levels under HS by Korotko et al. showed that most changes occurred after the HS treatment ended, with only a very small number occurring during the high-temperature treatment [24]. Statistical analyses revealed that changes in DNA methylation levels were associated mainly with coding regions [24]. Further, regulation of DNA (de)methylation had a positive effect on seed germination during plant development under HS [25]. BS-seq revealed that Copia elements were activated in response to the loss of DNA methylation in histone H1 mutants but not in heat-treated chromomethylase2 (cmt2) mutants. This finding suggests that the maintenance of DNA methylation under heat by H1 represses Copia elements [11].

Although several in-depth omics studies on plant HS responses have been conducted, regulatory differences, as well as gene expression differences, among different tissues of plants under HS remain poorly understood. In addition, there is a knowledge gap as to whether certain core genes, which play important roles in regulating heat responses in various tissues of plants, have different regulatory mechanisms in different tissues. HEAT SHOCK TRANSCRIPTION FACTOR *A1B* (HSF *A1B*), which is required early in the stress response for the transient expression of heat shock genes, encodes a putative transcription factor whose expression is not induced by heat and whose stable expression leads to the induction of HSPs. Overexpression of *A1B* in Arabidopsis not only promotes enhanced resistance to abiotic and biotic stresses but also affects development by causing a redistribution of biomass to reproductive structures at the expense of vegetative shoot growth, thereby improving seed yield [26,27,28]. The study of the model plant Arabidopsis is key to the exploration of temperature and thermal response mechanisms. *Arabidopsis thaliana* has been widely used as a model organism because of its small genome, rapid growth, short generation time, and high transformation efficiency. In addition, genes isolated from Arabidopsis can be used to identify homologs in many economic crops, including those associated with HS, which also has implications for other plant species [1].

Therefore, we examined in-depth the core HS genes with differential expression in Arabidopsis seed, leaf, and seedling tissues using multiomics approaches and explored the upstream transcription factors to elucidate their regulatory mechanisms. Further, the mechanistic differences between leaf tissue and the other two tissues were investigated to identify unique regulatory pathways and core regulatory genes in leaves. This information not only fills a knowledge gap in the exploration of differential regulatory mechanisms among tissues in Arabidopsis but also for the core HS genes found throughout plants. Finally, our work provides an important candidate marker gene for the study of HS mechanisms in plants and lays the foundation for targeted improvement of Arabidopsis and its resistance to adverse conditions.

## 2. Results

### 2.1. Transcriptome Advanced Analysis of Heat-Stressed Arabidopsis

**Figure 1:**
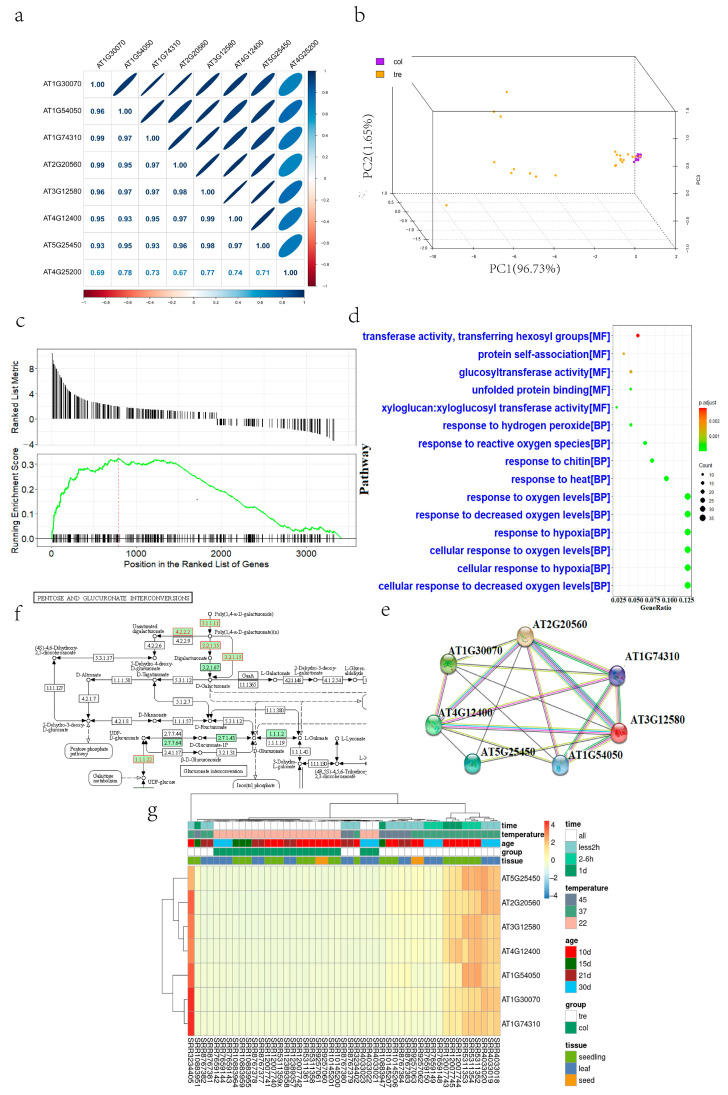
Differential gene expression in Arabidopsis. (**a**) Gene correlation matrix. (**b**) Col represents normal samples, and tre represents heat-treated samples. (**c**) Seed ath01100 GSEA analysis. (**d**) Leaf RNA GO enrichment analysis. (**e**) Gene interaction network. (**f**) Seed ath00040 pathway diagram, where circles represent metabolites involved in a pathway, boxes represent genomes and chemical reactions involved in the transition between metabolites, and green boxes represent chemical reactions and genomes unique to Arabidopsis in this pathway. (**g**) Heatmap of gene expression profiles of 49 libraries.

The intersection of DEGs between 49 libraries was obtained for the three tissues (seedlings, seeds, and leaves; Supplementary Figure S1a–c). After comparing the samples in pairs (Table S3), the intersection of the results was calculated. We obtained 8 DEGs, constructed a matrix of Pearson’s correlation coefficients among the nine genes, and developed a correlation heatmap (Figure 1a). We selected seven highly correlated genes, *AT1G30070, AT1G54050* (*HSP17.4B*), *AT1G74310* (*HSP101*), *AT2G20560* (*DNAJ*), *AT3G12580* (*HSP70-4*), *AT4G12400* (*HOP3*), and *AT5G25450*, for further study.

We then performed PCA on the 49 libraries using the expression levels of these 7 genes as variables. The PCA principal component analysis constructed from these seven genes can roughly distinguish between Arabidopsis under normal and heat stress conditions (Figure 1b). 

Further, after interaction network analysis, it was found that there were quite significant and diverse interactions among these 7 genes, suggesting that they might constitute a complex regulatory network with each other (Figure 1c). 

We compared different leaf samples (seed, seedling) and obtained the DEGs in the leaves (seed, seedling), then we did GO enrichment analysis for leaf DEGs, seed DEGs and seedling DEGs. WGCNA analysis (refer to the next section) showed that no gene set was significantly associated with stress temperature change, so we selected different tissue samples with stress times all between 2 and 6 h for analysis. To explore the regulatory mechanism of different tissues in response to heat stress, we selected 10 day old seedling samples treated at 37 °C for 3 h, 21 day old leaf samples treated at 45 °C for 5 m and 10 day old seed samples treated at 37 °C for 3 h for KEGG and GSEA analysis. The GO enrichment analysis showed that the three Arabidopsis tissues produced a different pattern of DEGs under HS conditions. The pathways in leaf tissue are mainly enriched in oxygen regulation-related pathways (Figure 1d, Supplemental Figure S1a,b). 

Differences in enrichment results were most obvious for leaf and seed tissues. DEGs in the leaf tissue were mainly enriched in the regulation of oxygen levels, whereas samples from DEGs in the seed tissue were mainly enriched in the regulation of various protein folding and refolding functions. In contrast, DEGs in the seedling tissue were enriched for protein folding and oxygen level regulation. Moreover, KEGG analyses exhibited the same trend (Supplemental Figure S1c,d), and enrichment result differences were most obvious in seeds and leaves. In contrast, seedlings were enriched in seed- and leaf-specific pathways. 

Then, we performed GSEA to determine the most significantly enriched KEGG pathways in leaf, seed, and seedling samples to obtain the significantly enriched core gene sets. Core gene sets were subsequently annotated onto enriched pathway maps (Figure 1e,f). Clearly, the core genome of Arabidopsis seeds is remarkably clustered into pentoses and glucuronate interconversion. 

Finally, we constructed a heat map to group Gene expression profiling with differentially expressed genes. The expression difference before and after HS is quite significant (Figure 1g).

### 2.2. Weighted Gene Co-Expression Network Analysis (WGCNA) of the Transcriptome

We first calculated a scale-free distribution topology matrix to determine the optimal β value (Supplemental Figure S2a) for WGCNA analysis using the expression level of all the co-annotated genes of the 49 Arabidopsis libraries as input data. A power β value = 16 was chosen for subsequent analysis. 

A co-expression matrix with Arabidopsis co-annotated genes was subsequently constructed, classifying 5000 genes from 17,887 into 20 modules (Supplemental Figure S2b). 

A systematic clustering tree was then constructed for the samples, and the corresponding conditional grouping module was added to determine the direction of subsequent analyses. We used temperature, duration of HS, and sample tissue type as grouping conditions to generate clearer clusters or groupings of sample tissue types (Supplemental Figure S2c). 

**Figure 2:**
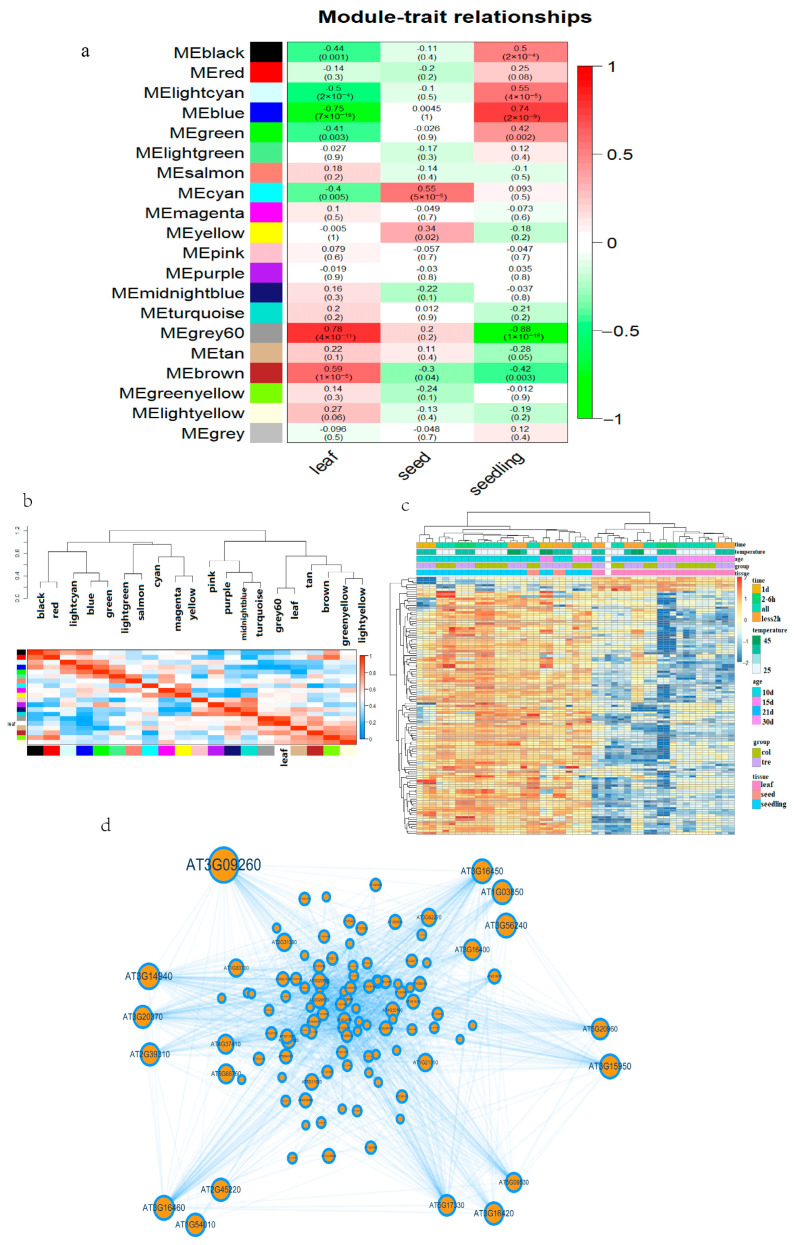
WGCNA analysis in Arabidopsis. (**a**) Heatmap of the correlation of gene modules with Arabidopsis tissues. (**b**) Correlation and clustering analyses of leaf tissue with whole gene clustering modules. (**c**) Gery60 module gene expression heatmap. Time represents heat stress treatment time, and ‘all’ represents no heat stress treatment. (**d**) Grey60 module gene co-expression network, where the size of the circle represents the number of intergenic interactions, and high and low correlation coefficients are indicated by the thickness of the line.

To quantify the specific correlation between individual gene modules and each condition of a sample, we constructed a correlation coefficient heatmap of gene clustering modules and sample characteristics. The correlation heatmap constructed with temperature and HS time as the sample grouping characteristics did not show high correlation coefficients (Figure 2a, Supplemental Figure S2d,e). 

For the correlation heatmap constructed with sample tissue type as the grouping characteristic, there were obvious differences among gene modules. Specifically, the correlation coefficient between leaf tissue samples and the gene module grey60 was 0.78 with a significance of 4 × 10^−11^. This finding is consistent with the sample systematic clustering results. Therefore, genes from the grey60 module of leaf samples were selected for the next analysis. First, we performed a correlation comparison of the 112 genes of this module for leaf samples (Supplemental Figure S2f). 

Clearly, these genes were highly correlated not only with their corresponding modules but also with their corresponding traits, further illustrating the value of in-depth analyses. We then constructed a gene heatmap for all genes across all samples, selecting 400 genes for visualization (Supplemental Figure S2g). 

Subsequently, we selected leaves as the trait and constructed a feature module correlation heatmap. The cluster with the leaf phenotype was found to be the closest, in terms of distance, to grey60 (Figure 2b). The 112 genes inside the extraction module grey60 were used to construct a gene expression level grouping heatmap (Figure 2c).

Finally, we constructed a co-expression network map of these 112 genes. There was a clear difference between leaf tissue and the other tissues in the grouping (Figure 2d). Genes *AT3G09260* (*BGLU23*), *AT3G14940* (*PPC3*), and *AT3G15950* (*NAI2*) had higher degrees (the number of genes connected in the co-expression network) and, thus, are likely to be core genes among the gene modules.

### 2.3. Regulation Patterns of Transcription Factor A1B in Arabidopsis

**Figure 3:**
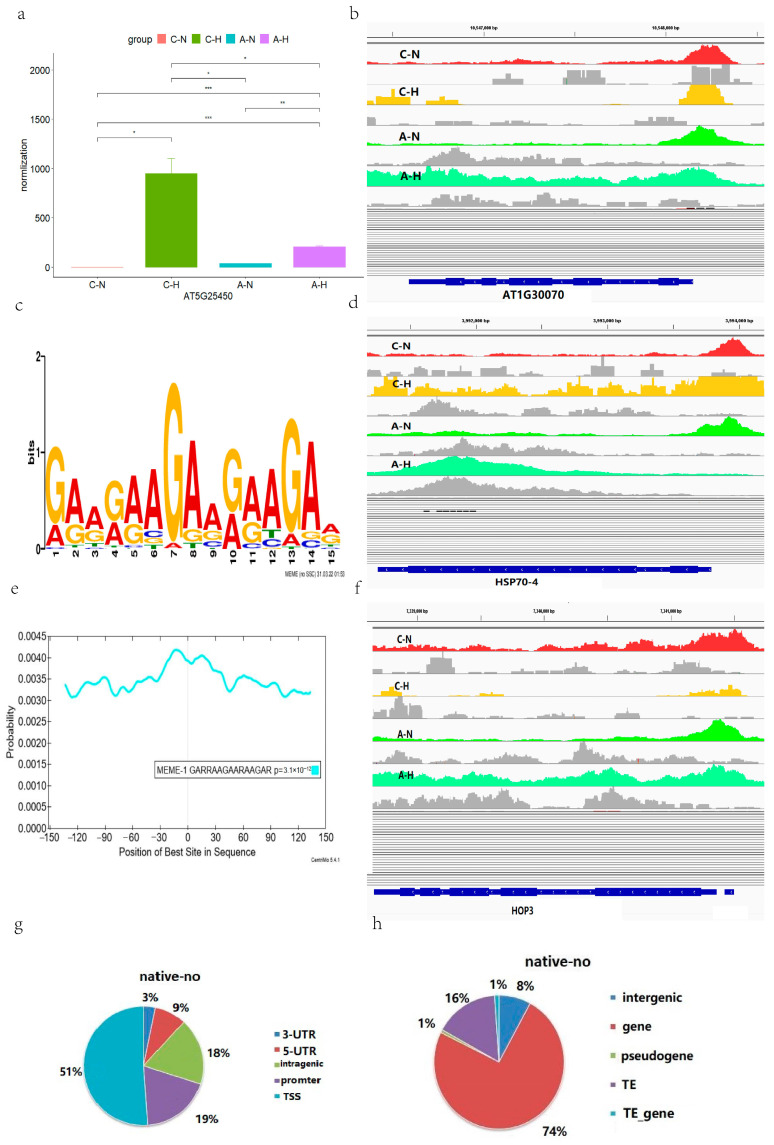
*A1B* Chip-seq analysis in Arabidopsis. (**a**) *AT5G25450* expression. “*” Represents *p* ≤ 0.05, “**” represents *p* ≤ 0.01, and “***” represents *p* ≤ 0.001. (**b**) *AT1G30070* peak profile. Red represents wild-type Arabidopsis, yellow represents the wild-type heat stress group, light green represents the overexpression *A1B* control group, and dark green represents the overexpression *A1B* heat stress group. Wild type at normal temperature (C-N). Wild type under high temperature stress (C-H). *A1B* overexpression at normal temperature (A-N). *A1B* overexpression under high temperature stress (A-H). (**c**) MEME analysis of motifs identified in the ChIPseq peaks. The larger the base font, the more likely the site is that base. (**d**) *HSP70-4* peak profile. (**e**) Subject location likelihood prediction. Probability curves for motif enrichment at the center of input sequences. (**f**) *HOP3* peak profile. (**g**) Peak types in wild-type controls. (**h**) Peak types in wild-type controls.

The seven genes (*AT1G30070*, *HSP17.4B*, *HSP101*, *DNAJ*, *HSP70-4*, *HOP3* and *AT5G25450*) identified from the differential genetic analysis, we intended to further explore their mechanisms of action under HS, we collected chip-seq and RNA-seq sample data from the GEO database to explore the regulation of seven genes by the *A1B*. We found that the expression levels of the seven genes were significantly different in the normal seedling sample, the sample of seedlings overexpressing *A1B*, the sample of seedlings with HS, and the sample of seedlings with HS overexpressing *A1B*; the trend was consistent (Figure 3a, Supplemental Figure S3a–f).

The seven genes were compared in four different types of seedling samples. The expression of these genes was lowest in the wild-type seedling sample. The expression of these genes was highest in the wild-type seedling sample after HS, and expression of all these genes was significantly elevated in the Arabidopsis sample with the *A1B* overexpression compared with the wild-type Arabidopsis without HS. However, in heat-stressed *A1B* overexpression Arabidopsis samples, the expression amounts of these genes were higher than that in non-heat-stressed Arabidopsis but lower than that in heat-stressed wild-type Arabidopsis. This finding is consistent with that of Albihlal et al. [29]. 

In addition, six of the genes were found by chip-seq to occur in the annotation results of wild-type Arabidopsis, while *AT1G30070* did not appear in the annotation results. Annotation results for these seven genes were found in *A1B*-mutated (overexpressed) normal Arabidopsis samples and wild-type heat-stressed Arabidopsis samples. However, only *AT3G12580* and *AT4G12400* were present in the annotation of the *A1B*-mutated (overexpressed) heat-stressed Arabidopsis samples (Figure 3b,d,f, Supplemental Figure S3g–j). 

Further, we performed motif analysis of the binding sites and found that there was no change in the conserved binding sequences before and after high-temperature stress (Figure 3c). In addition, conserved motifs are also ubiquitously located in the center of the sequenced sequence (Figure 3e).

Finally, we performed a statistical analysis on the methylation position and the type of site where methylation was located; the three different types of methylation shared similar patterns. The major methylation sites were in the 3′ untranslated region (UTR) of the gene, the transcriptional start sites (TSS), and the promoter (Figure 3g,h, Supplemental Figure S3k–p).

### 2.4. MicroRNA Regulatory Pathways in Heat-Stressed Arabidopsis

**Figure 4:**
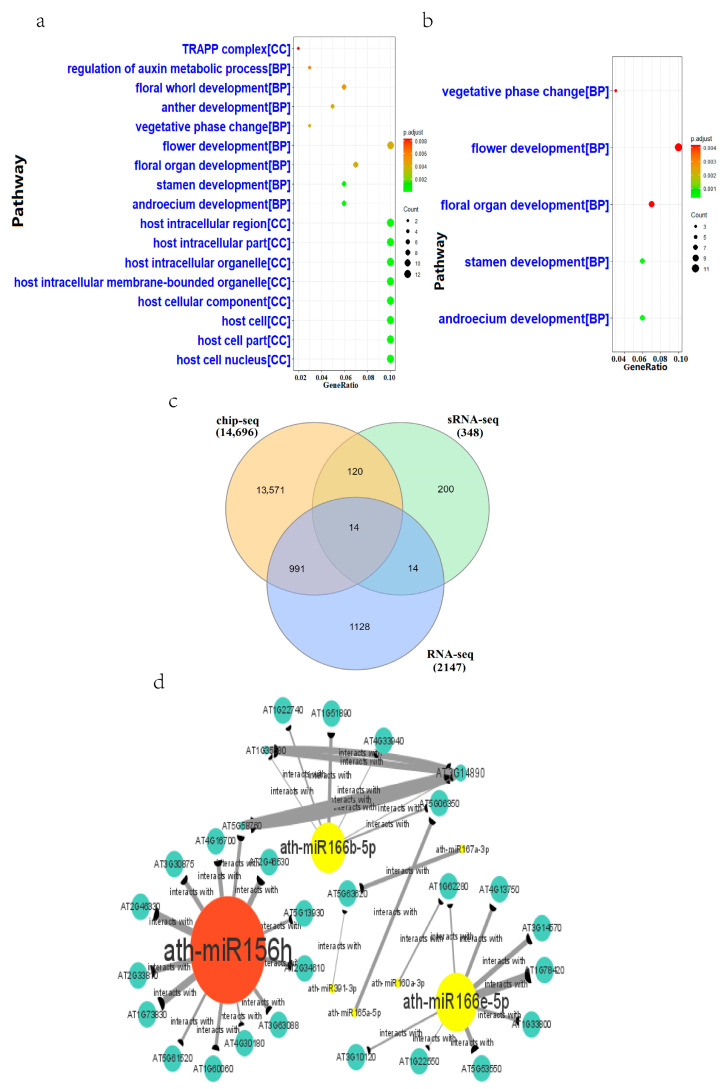
Differential microRNA analysis in Arabidopsis. (**a**) 15 day old heat-stressed 0.5 h Arabidopsis seedlings. Differential microRNA target gene GO enrichment analysis. (**b**) 15 day old heat-stressed 6 h Arabidopsis seedlings. Differential microRNA target gene GO enrichment analysis. (**c**) The common differential genes identified from three omics analyses. (**d**) CeRNA network. Circle size represents the number of acting target genes, and high and low correlation coefficients are indicated by the thickness of the line. Red represents upregulated microRNAs, and yellow represents downregulated microRNAs.

We collected seedling samples for demiRNA analysis with two treatment conditions of 0.5 h and 6 h, and eight demiRNAs in the 0.5 h group were selected; one was upregulated, and seven were downregulated. Thirteen demiRNAs were selected from the 6 h group; three were upregulated, and ten were downregulated. GO enrichment analysis of the target genes predicted by the upregulated and downregulated demiRNAs (Figure 4a,b) showed that the predicted target genes of the upregulated demiRNAs were significantly enriched in the heat-stressed Arabidopsis samples.

We subsequently intersected predicted target genes of the 0.5 h group and the 6 h group. The resulting genes were sequentially intersected with seedling differential genes from RNA-seq, resulting in a final dataset of 28 genes (Figure 4c). Therefore, we inferred that these 28 genes were regulated by miRNAs under HS conditions. Next, we constructed a competing endogenous RNA (ceRNA) network utilizing 7 demiRNAs and 28 differential target genes (Figure 4d). By reviewing the literature, upregulated miRNAs and downregulated miRNAs were identified, with ath-miR156h, ath-miR166b-5p, and ath-miR166e-5p identified as core regulatory miRNAs.

### 2.5. DNA Methylation Regulation Patterns Exist in Heat-Stressed Arabidopsis

**Figure 5:**
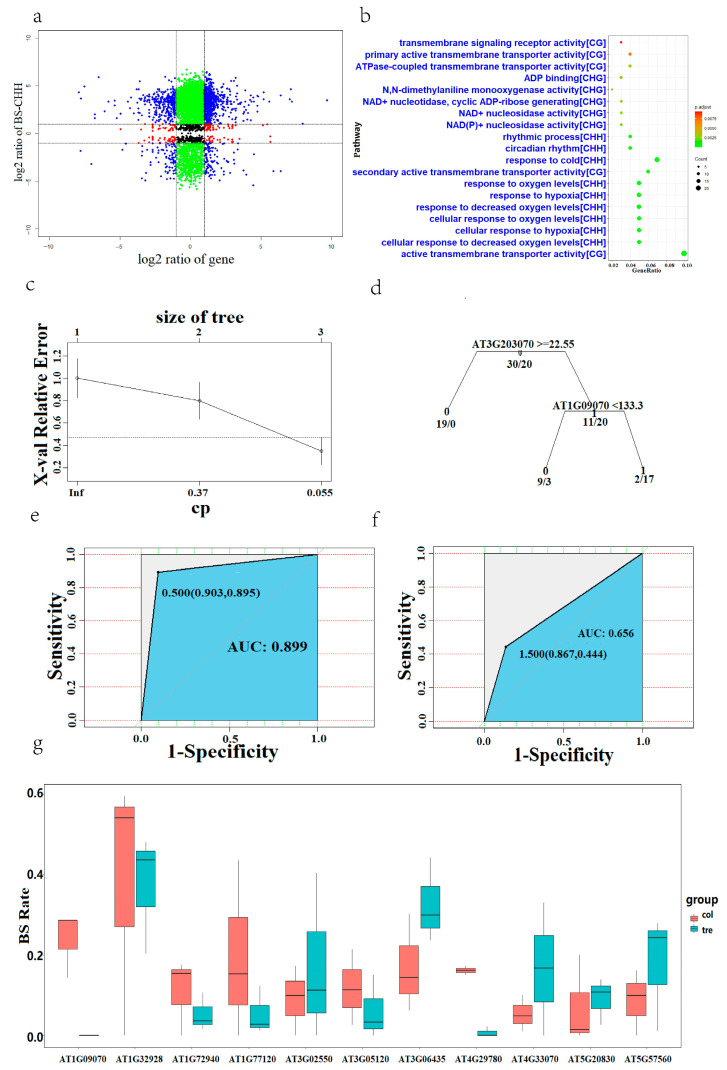
BS-seq differential analysis in Arabidopsis. (**a**) Nine quadrant diagram of genes differentially methylated by CHH. Divide by log2FC values of 1 and −1. (**b**) GO enrichment analysis of differentially methylated genes. (**c**) Evolution of the decision tree, where the bottom x-axis is the CP value, the y-axis is the relative error, and the top x-axis is the size of the tree. The dotted line is the upper limit of the standard deviation. (**d**) Decision tree. (**e**) Training set ROC curve. (**f**) Test set ROC curve. (**g**) Methylation degree statistics. The horizontal axis is the gene involved in the regulation of oxygen levels, and the vertical axis is the methylation proportion at that site.

We collected methylation sample data of Arabidopsis leaves from the GEO database to explore the expression patterns of genes in Arabidopsis leaves under HS. DNA methylation is classified into three types, CG, CHG, and CHH, and we screened differentially methylated sites and their corresponding annotations for 17,790, 17,590, and 14,645 genes, respectively. The annotated genes were then intersected with RNA-seq demiRNAs. A nine quadrant plot with log_2_FC as the coordinate axis was then constructed, and genes in the first and ninth quadrants were selected. Of these genes, 320, 441, and 479 were differentially methylated genes of the CG, CHG, and CHH types, respectively (Figure 5a, Supplemental Figure S4a,b). These genes are presumed to be regulated by changes in methylation levels under stress. The annotated intersection DEGs were subsequently subjected to GO enrichment analysis (Figure 5b). 

We found that the enriched pathways identified by our CHH screen were similar to pathways identified by RNA-seq, involving 479 genes. Among these genes, 17 were enriched in pathways involved in the regulation of oxygen levels. Subsequently, we extracted the 12 key genes from these 17 genes and merged them with the 12 core regulatory genes in the formed gery60 module co-expression network for a joint analysis to explore the core regulatory genes that distinguish Arabidopsis leaves from other Arabidopsis tissues in response to HS. These 12 genes were *AT1G09070* (*ATSRC2*), *AT1G54010* (*GLL23*), *AT2G39310* (*JAL22*), *AT2G45220* (*ATPME17*), *AT3G06435, BGLU23, PPC3, NAI2, AT3G16420* (*PBP1*), *AT3G16450* (*JAL33*), *AT3G16460* (*JAL34*), and *AT3G20370*. A classification model was constructed using a training set decision tree. When the decision tree hierarchy was 3, the cross-validation error no longer dropped, and the cross-validation error was a minimum of 0.35 (Figure 5c). 

The decision tree model shared two genes, *AT1G09070* and *AT3G2037* (Figure 5d). One gene, AT1G09070, was from the 17 genes enriched in the regulation of oxygen levels, and the other gene, AT3G2037, was from the co-expression network constructed with WGCNA that was highly correlated with leaf tissue. The AUC value of the decision model on the training set was 0.899, with a sensitivity of 0.865 (Figure 5e). For the decision model on the validation set, the AUC value was 0.656, and the sensitivity was 0.444 (Figure 5f). 

Both the AUC value and sensitivity showed a clear decrease for the validation set. Finally, we combined methylation data of 17 genes associated with oxygen-regulated pathways in 21 day old Arabidopsis leaf samples (from SRR10401148- SRR10401150) with those of samples with 24 h of HS to show CHH methylation changes of these genes before and after HS. We utilized replicates to remove likely false-positive results and found that 11 were informative about oxygen level conditions; the resulting *AT1G09070* gene was in the decision tree (Figure 5g). The gene AT1G09070 had the greatest difference in the degree of CHH methylation, which confirmed the results of the decision tree model construction.

## 3. Discussion

### 3.1. Core Genes and Tissue Specificity in Heat-Stressed Arabidopsis

The morphological and functional integrity of the cell depends on the balance of most, if not all, encoded proteins, also known as proteostasis, which includes control of synthesis, intracellular sorting, folding, protein function, and degradation [30]. This includes cytosolic proteins as well as proteins in other cellular compartments. All organisms need to maintain proteostasis in an optimal state of growth and development under normal and stressful environmental conditions. High temperature activates the transcription of HS-induced genes, triggering the cellular heat stress response (HSR), ultimately leading to an increase in the concentration of incorrectly folded and aggregated proteins [6]. The most prominent examples are the classical HSPs encoding molecular chaperones [6,31].

Through RNA-seq, we obtained 8 DEGs, which may play important roles in HS in various Arabidopsis tissues. We also found seven genes, *AT1G30070*, *HSP17.4B*, *HSP101*, *DNAJ*, *HSP70-4*, *HOP3*, and *AT5G25450*, which all function as molecular chaperones and play heat regulatory roles in Arabidopsis leaf, seedling, and seed tissues, and express Hsp20-like chaperonin superfamily proteins. *AT1G30070* [32], *HSP 101* [33], *DnaJ* [34], *HSP 70-4* [35], and *HOP3* stress-inducible protein [36], respectively. 

The PCA constructed from these seven genes can roughly distinguish samples before and after HS. Among them, at room temperature, they are concentrated together, while under heat stress, Arabidopsis is more dispersed. We speculate that the expression of these seven genes will undergo changes under different HS conditions.

Therefore, we conclude that these genes may play a core role or be related to core genes in the gene regulation of the HS response and may play different roles in early and late stress. Further, there were quite significant and diverse interactions between the proteins expressed by these genes, which suggested that they might constitute some complex interacting regulatory mechanisms with each other. 

Samples from Arabidopsis leaf tissue responded to HS mainly by regulatory changes at the oxygen level, while samples from seed tissue responded to HS mainly through regulation of protein folding and refolding reactions.

After annotating the core enriched genes of the ath00040 pathway in seeds, we found three specific metabolic pathways may respond to HS, which involve the conversion of 1,4-α-D-galacturonate to unsaturated digalacturonate, 1,4-α-D-galacturonate to D-galacturonate, and 1,4-α-D-galacturonate to digalacturonate (Figure 1f).

### 3.2. Altered Binding Profiles of Transcription Factor A1B in Heat-Stressed Arabidopsis

The transcription of some heat shock protein (HSP) genes is regulated by mild and elevated temperatures and can even act as a “molecular thermometer” [37]. However, these molecular chaperones, such as members of the heat shock transcription factors (HSFs), are mostly associated with the HS response [37].

Using chip-seq, we found that seven genes, *AT1G30070* [38], *HSP17.4B*, *HSP101*, *DNAJ*, *HSP70-4*, *HOP3*, and *AT5G25450*, were regulated by the *A1B* HSF and the binding profiles of *A1B* were changed in response to HS, affecting the expression levels of these genes. That is to say, the expression of the *A1B* gene does not change under heat stress, while the binding profiles of the *A1B* heat shock transcription factor were changed in response to heat stress, which affected the expression levels of these seven genes.

Among these genes, *AT5G25450* encodes a cytochrome bd ubiquinol oxidase that plays a role in salt stress, ABA stress, and oxidative stress [39] and has now been shown to be responsive to HS and regulated by *A1B*. 

The results illustrate that the binding profile of *A1B* is altered under conditions of heat stress and that the binding profile of overexpressed *A1B* also exhibits distinct differences compared with *A1B* in the normal state. These results of binding profile changes are consistent with the findings of Albihlal et al. [29]. Finally, motif analysis of our chip-seq results for four different types of samples found that the binding sequence of *A1B* was not affected by HS and overexpression, so the transcription factor binding profile changes were not caused by the binding sequence changes, but the specific regulatory mechanisms remain to be explored. 

In summary, we found that seven genes were regulated by the *A1B* HSF and that the binding profile of *A1B* changed in response to HS. In the *A1B* overexpressed heat-stressed Arabidopsis samples, only *HSP70-4* and *HOP3* were within their binding profile. This change in the conjugation lineage might be responsible for the lower expression levels of these genes in the heat-stressed Arabidopsis samples with *A1B* overexpression than in the wild-type ones. 

In addition, the significantly higher expression of these genes in the unstressed *A1B* overexpressed Arabidopsis samples compared with the wild-type Arabidopsis samples that were not subjected to HS may be due to either the promotion effect caused by the increased binding ability of overexpressed *A1B* to the target genes or the indirect promotion effect caused by the changed *A1B* binding profile. This intrinsic mechanism remains unclear.

### 3.3. Core microRNAs in Heat Stressed Arabidopsis

Through sRNA-seq, the floral organ development and floral whorl development pathway in 0.5 h heat stress-treated Arabidopsis were suggested to play an important role in the HS response, while flower development in 6 h HS-treated Arabidopsis might also play an important role in the stress response. Combined with the above data and GEO database samples, we found that two miRNAs, ath-miR156h and ath-miR166b-5p, respond to high-temperature stress and promote sustained expression of HS-responsive genes in Arabidopsis [40]. We have verified this conclusion once again using bioinformatics methods. Under normal conditions, high temperatures are lethal to plants. Heat stress memory can allow plants to acquire heat tolerance to withstand elevated temperatures and maintain them to non-elevated temperatures for several days [41].

Moreover, we found that ath-miR156h and ath-miR166b-5p [42] from Arabidopsis seedlings may play a core regulatory role in response to HS or be related to core regulation in HS reactions. We speculate that the upregulated ath-miR156h mainly acts on ARF11 auxin response factor 11, BEE3 BR enhanced expression 3, HLH4, and SPL3 squamosa promoter binding protein like 3 to inhibit its expression on HS and affect transcription factor activity, thereby exerting regulatory effects. Downregulated ath-mir166b-5p may be mainly mediated by targeting dead/death box RNA helicase family proteins, damaged DNA binding proteins, and genes such as ATP-dependent DNA helicase Q-like 4A/4B to promote these genes to play an impact on DNA damage repair in response to HS. However, many aspects of this study require a more thorough investigation. 

For example, gene expression of *A1B*-overexpressing heat-stressed Arabidopsis leaves was significantly lower than that of wild-type heat-stressed Arabidopsis leaves, the differential miRNA ath-miR166e-5p [42] in Arabidopsis HS response, and whether the 17 enriched genes involved in the regulation of oxygen levels were involved in the HS response through methylation degree changes under HS treatment at different temperatures and times remain unknown. 

This is also the first time that two miRNAs, ath-miR166b-5p and ath-miR166e-5p, have been identified in response to HS and may have core regulatory roles. In addition, the DEGs revealed by RNA-seq above were not the targets of the demiRNAs. Clearly, there are significant differences in the ways in which different genes are regulated in response to HS.

### 3.4. CHH Methylation in Response to Heat Stress in Arabidopsis

In Arabidopsis thaliana, global genome-wide levels of 24% CG, 6.7% CHG, and 1.7% CHH methylation were observed [43]. DNA methylation in plants mainly occurs at transposons and other repetitive DNA elements [44]. The establishment, maintenance, and removal of DNA methylation require the participation of many different proteins. CHG methylation requires the plant-specific DNA methyltransferase CHROMOMETHYLASE 3 (CMT3) for its maintenance [10].

BS-seq found that, at high temperatures, plants may be able to regulate oxygen levels by altering the degree of CHH methylation. We screened 17 genes by a nine-quadrant plot, then constructed a decision tree model by combining the co-expression network results from WGNCA, and finally obtained two genes, *ATSRC2* [45] and *AT1G09070*, in which *AT1G09070* plays a role in protein targeting to the vacuole [46]. It is also the first time that these two genes may affect changes in their methylation levels in their response to HS. These two genes likely play important roles in the short-term (24 h) HS response of 21 day old Arabidopsis leaves. 

Finally, the predictive power of two genes, *ATSRC2* and *AT1G09070*, dropped significantly in the validation set of the decision tree due to sample size limitation. The number of samples for both the training and validation sets was subsequently changed slightly, and the constructed decision tree was found to be unchanged, and the accuracy of the prediction only changed slightly. These results suggest that *ATSRC2* and *AT1G09070* may play unique and important roles in the leaf senescence of heat-stressed Arabidopsis. Therefore, the real predictive ability of the two genes *ATSRC2* and *AT1G09070* and their roles in the different prolonged HS responses of different aged Arabidopsis leaves need to be further investigated.

## 4. Materials and Methods

### 4.1. Screening of Differential Genes in the Transcriptome of Heat-Stressed Arabidopsis thaliana

Forty-nine Arabidopsis HS RNA-seq libraries (Table S1) were downloaded from the Gene Expression Omnibus (GEO) database (https://www.ncbi.nlm.nih.gov/geo/, accessed on 30 April 2022). Four were seed libraries, 24 were seedling libraries, and 20 were leaf libraries. The original data file was changed into a fastq file after quality control using fastq-dump and Trim Galore! (version 3.7). HISAT2 (version 2.2.1) [47] was used to align the sequences, and a program written by our laboratory was used to determine gene expression from the results of the HISAT2 analysis. Finally, edgeR [48] was used to calculate differential gene expression. The threshold of DEGs was log2FC > 2 or <−2, RPM > 1, and *p*-value < 0.05. The resulting DEGs were used in downstream analyses.

The RNA-seq section of Table S1 lists all the 49 libraries used in our analysis in this section [11,14,15,21,29,49,50,51]. Table S3 records the number of differential genes compared between groups. Supplementary Material S1 provided the list of genes for comparison between groups. We recorded the SRA number, age, treatment conditions, and so on for each library. “tre” represents heat stress, while “col” represents normal temperature. To facilitate comparison between libraries, we also grouped each batch of biological replicates. Libraries from the same group were analyzed for gene expression.

### 4.2. Weighted Gene Co-Expression Network Analysis (WGCNA) of Transcriptome

The relationships between phenotypic features and gene expression quantities of the 49 Arabidopsis libraries were analyzed using the WGCNA package (version.1.71) [52] in R. A scale-free distribution topology matrix was calculated to determine the optimal β values. A power β value of 16 was selected for subsequent analysis. Subsequently, the correlation coefficients among genes, modules, and traits, as well as the significance matrix, were obtained. Enrichment analysis was performed targeting gene modules significantly associated with the trait.

### 4.3. Enrichment Analysis of Heat Stress in Arabidopsis

For genes obtained in this study, gene ontology (GO) enrichment analysis, Kyoto Encyclopedia of Genes and Genomes (KEGG) enrichment analysis, and gene set enrichment analysis (GSEA) was performed using the clusterProfiler package in R (version 4.2.2) [53]. For GO enrichment analysis, the arguments were set as follows: pAdjustMethod = BH, pvalueCutoff = 0.01, and qvalueCutoff = 0.05. For KEGG analysis, the gseKEGG function was used, and for GSEA analysis, the GSEA function was used.

### 4.4. PCA and String of Core Genes

PCA analysis was carried out by using the prcomp function of R, and the libraries were dimensionally reduced with the expression of the 7 genes we selected as the input attribute of each library. Gene interaction network was constructed on an online website (https://string-db.org/, accessed on 20 May 2022) [54].

### 4.5. Chip-Seq Was Used to Explore Transcription Factor Changes in Response to Heat Stress

Sixteen Arabidopsis HS chip-seq libraries were downloaded from the GEO database. The original data file was changed into a quality-controlled fastq file using fast-dump and Trim Galore! Bowtie (version 7.3.1) [55] was used to align sequences, and SICER (version 1.1) [56] was used to call peaks. Peaks were visualized with the Integrative Genomics Viewer (IGV; version 2.12.3) [57]. Motif analysis was performed to extract the peaks corresponding genomic sequences and search for similar sequences using MEME (version 5.4.1) [58], and differential chip regions (DCRs) were annotated using SICER. Results were subjected to downstream analyses.

The Chip-seq section of Table S1 lists all libraries used in our analysis in this section [29]. There are 4 types of conditions: wild type at normal temperature (C-N); wild type under high temperature stress (C-H); *A1B* overexpression at normal temperature (A-N); *A1B* overexpression under high temperature stress (A-H). Supplementary Material S1 provided the peak calling result and gene expression list of Chip-seq.

### 4.6. Exploration of Core microRNAs as Well as Their Regulatory Pathways

We directly downloaded the result file of the miRNA expression matrix generated from the processing of the sequence read archive (SRA) original file (SRR1848803-11) through the GEO database, including that 2 heat stress conditions of 0.5 h as well as 6 h. Differentially expressed miRNAs with log2FC > 2 or <−2, RPM > 1, and *p*-value < 0.05 were selected for analysis. All target genes of the differentially expressed miRNAs were predicted using a forecast website (https://www.zhaolab.org/psRNATarget/, accessed on 20 May 2022) [59]. Finally, the regulation mechanism of miRNAs in Arabidopsis was investigated. Single-cell RNA (scRNA) network maps of Arabidopsis were constructed with Cytoscape using the intersection values of differentially expressed miRNAs and target genes and the intersection values of target and differential genes (version 3.9.1) [60].

The sRNA-seq section of Table S1 lists all libraries used in our analysis in this section [13]. Supplementary Material S1 provides a list of differential gene expressions of microRNAs.

### 4.7. Probing Patterns of Methylation Regulation

Six Arabidopsis HS BS-seq libraries were downloaded from the GEO database. The original data file was converted into a fastq file after quality control using fastq-dump and “Trim Galore!”. A result file containing the methylation sequence contexts CG, CHG, and CHH (where H is A, C, or T) for differential methylation regions (DMRs) was obtained using BS-Seeker2 (version 2.1.8) [61] and Bowtie (version 7.3.1) [55]. The DMRs of each mutant were defined by comparing their methylation levels in each cytosine context with those of 3 independent wild-type samples [62]. The methylation percentage of each site before and after heat treatment was determined to obtain the log2FC value for the subsequent 9 quadrant diagram and statistical analysis.

The BS-seq section of Table S1 lists all libraries used in our analysis in this section [24]. Supplementary Material S1 shows the methylation analysis results of CHH, CHG, and CG.

### 4.8. Search for Core Regulatory Genes Using Decision Trees

In addition to the 49 library gene expression data that we calculated as a result of our lab’s script above, we collected another 25 Arabidopsis sample gene expression data from the geodatabase (Table S1). An expression matrix containing 74 datasets and 24 genes was created. The expression matrix was divided into a training set containing 50 datasets and a validation set containing 24 datasets. Decision trees were constructed using the rpart package in R (version 4.1.16) [63] and appropriate CP, nsplit, and xstd values were obtained using the results of the printcp function. A decision tree was subsequently created. Finally, the ROC function from the pROC package in R (version 1.18.0) [64] was used to compute the receiver operating characteristic curve (ROC).

The 25 additional datasets section of Table S1 lists the supplementary libraries used for machine learning analysis [65,66,67,68,69,70]. Supplementary Material S1 provides the list of differential gene expressions provided.

## 5. Conclusions

Our results suggested that the genes *AT1G30070*, *HSP17.4B*, *HSP101*, *DNAJ*, *HSP70-4*, *HOP3*, and *AT5G25450* may play a central role in gene regulation in response to HS. Among these genes, *HSP17.4B*, *HSP101*, *DNAJ*, *HSP70-4*, and *HOP3* act as molecular chaperones and are members of the HSP family, playing a role in promoting appropriate protein folding in response to HS.

We infer that response to HS in seeds may be related to three key sugar metabolism reactions: the conversion of D-galacturonic acid to unsaturated galacturonic acid, galacturonic acid, or D-galacturonic acid.

*AT1G30070*, *HSP17.4B*, *HSP101*, *DNAJ*, *HSP70-4*, *HOP3*, and *AT5G25450* are regulated by *A1B*, and the binding spectrum of *A1B* will change under HS conditions. The expression levels of these genes are significantly lower in heat-stressed Arabidopsis samples with *A1B* overexpression than in wild-type samples, potentially owing to the change in the conjugation spectrum.

Two miRNAs in Arabidopsis seedlings, ath-miR156h and ath-miR166b-5p, may play a central regulatory role in response to HS.

*ATSRC2* and *AT1G09070* probably play an important role in the short-term (24 h) HS response of 21 day old Arabidopsis leaves.

## Data Availability

All raw sequencing data were downloaded from a public database. The detailed info can be found in Supplementary Table S1. The gene name refers to Supplementary Table S2.

## Supplementary Materials

The following supporting information can be downloaded at: https://www.mdpi.com/article/10.3390/ijms241311081/s1.
