# Supplementary material for: Multiomics Reveals the Regulatory Mechanisms of Arabidopsis Tissues under Heat Stress
Source: Int J Mol Sci. 2023 Jul 4;24(13):11081. doi: 10.3390/ijms241311081 (PMC10341750; doi:10.3390/ijms241311081)
Supplement: Supplementary file 1 [file ijms-24-11081-s001.zip › supplementaryFigureLegend.pdf]

Additional files

1. Supplementary table S1

List of NCBI SRA libraries used in this study.

2. Supplementary table S2

List of the gene name.

3. Supplementary table S3

Number of up-down genes per group.

4. Supplementary material S4.zip

RNAseq: Comparison list of genes for each group and Venn diagram of differential genes

Mirna: MicroRNA differential analysis list and up-down microRNA list

Chipseq: Chipseq annotation results under each condition

Bs-seq： Annotated results of three types of methylation mutations

25 additional datasets: Gene expression list of 25 supplementary libraries

5. Supplementary figure S1

sFig. 1 Differential gene analysis in Arabidopsis. (a) Seedling RNA GO enrichment analysis. (b) Seed RNA GO enrichment analysis. (c) Seedling RNA KEGG enrichment analysis. (d) Seed RNA KEGG enrichment analysis.

6. Supplementary figure S2

sFig. 2 WGCNA analysis in Arabidopsis. (a) Beta of scale-free distribution. The horizontal axes indicate the weight parameter β. The vertical axis of the left plot represents the square of the log (k) versus log (P (k)) correlation coefficient in the corresponding network. The vertical axis of the right plot represents the average of all gene adjacency functions in the corresponding gene module. (b) Gene system clustering and modularity. The upper section is the gene phylogenetic clustering tree and the lower section is the gene module. (c) Cluster visualization of samples. Red is Arabidopsis seeds, blue is leaves, and green is seedlings. (d) Heatmap of the correlation of gene modules with time of heat shock. (e) Heatmap of the correlation of gene modules with temperature. (f) Correlation analysis between gery60 module genes and leaf tissue. (g) 400 gene expression heat maps from each module were randomly selected

7. Supplementary figure S3

sFig. 3 A1b Chip-seq analysis in Arabidopsis. (a) AT1G30070 expression. (b) AT1G54050 expression. (c) AT1G74310 expression. (d) AT2G20560 expression. (e) AT3G12580 expression. (f) AT4G12400 expression. (g) AT2G20560 peak profile. (h) AT5G25450 peak profile. (i) AT1G54050 peak profile. (j) AT1G74310 peak profile. (k) Peak types in wild type heat stress. (l) Peak positions in wild type heat stress. (m) Peak types in overexpression A1B control. (n) Peak positions in overexpression A1B control. (o) Peak positions in overexpression A1B heat stress. (p) Peak types in overexpression A1B heat stress.

8. Supplementary figure S3

sFig. 4 BS-seq differential analysis in Arabidopsis. (a) Nine quadrant diagram of genes differentially methylated by CG. (b) Nine quadrant diagram of genes differentially methylated by CHG.
