# Supplementary figures and images for: Multiomics Reveals the Regulatory Mechanisms of Arabidopsis Tissues under Heat Stress
Source: Int J Mol Sci. 2023 Jul 4;24(13):11081. doi: 10.3390/ijms241311081 (PMC10341750; doi:10.3390/ijms241311081)

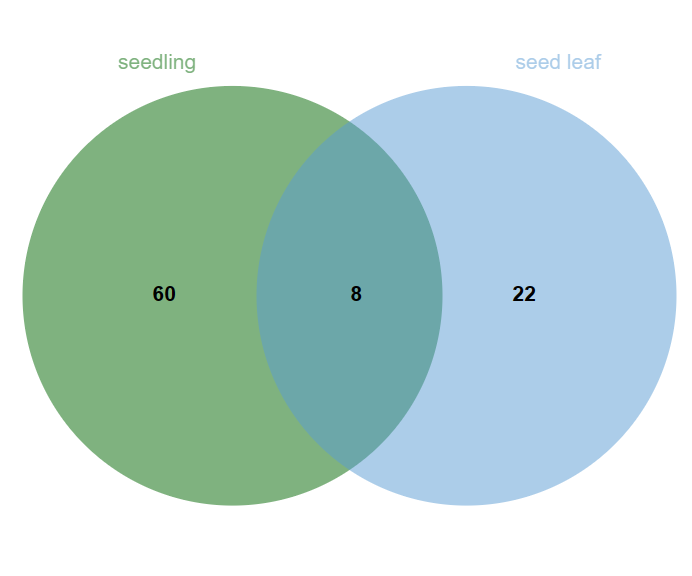

Supplement: Supplementary file 1 [file ijms-24-11081-s001.zip › 3tissuevenn.png]

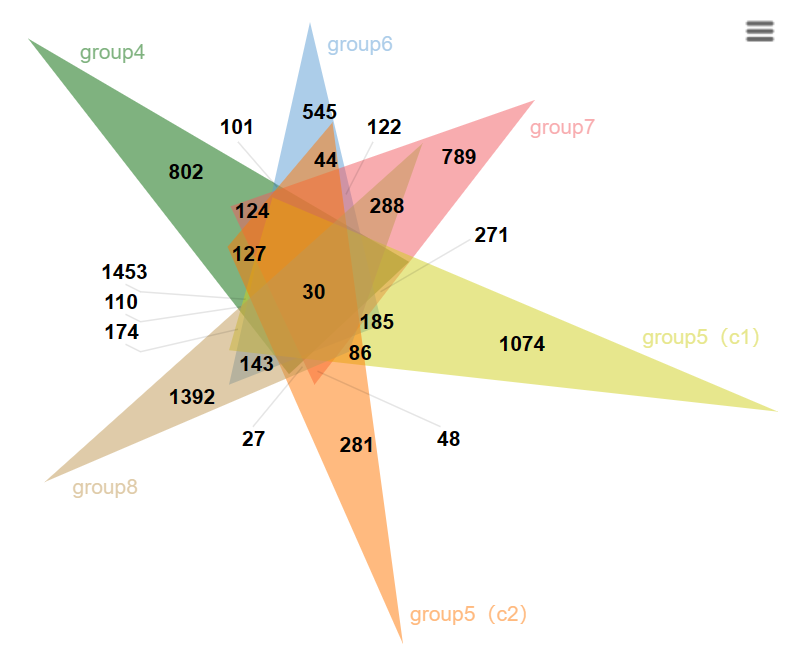

Supplement: Supplementary file 1 [file ijms-24-11081-s001.zip › seed-leafvenn.png]

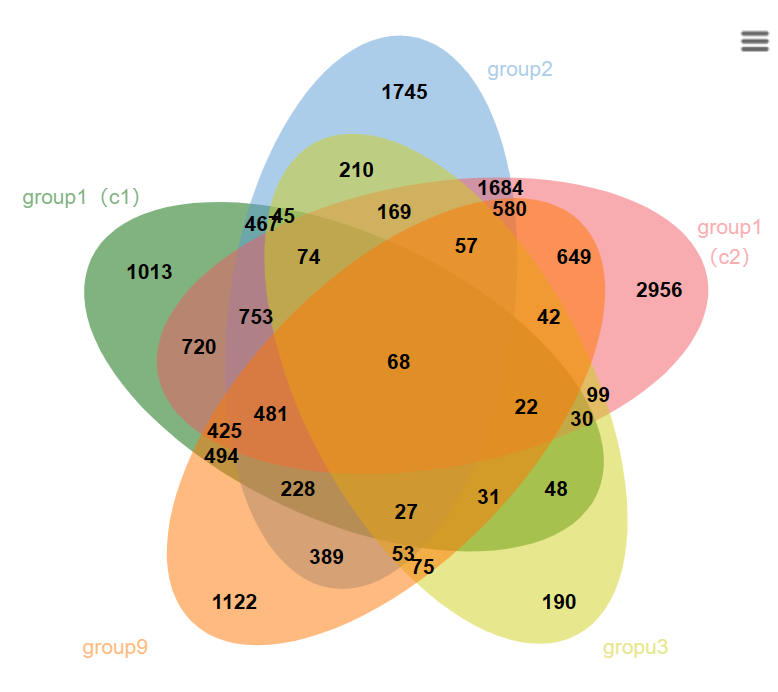

Supplement: Supplementary file 1 [file ijms-24-11081-s001.zip › seedlingvenn.png]

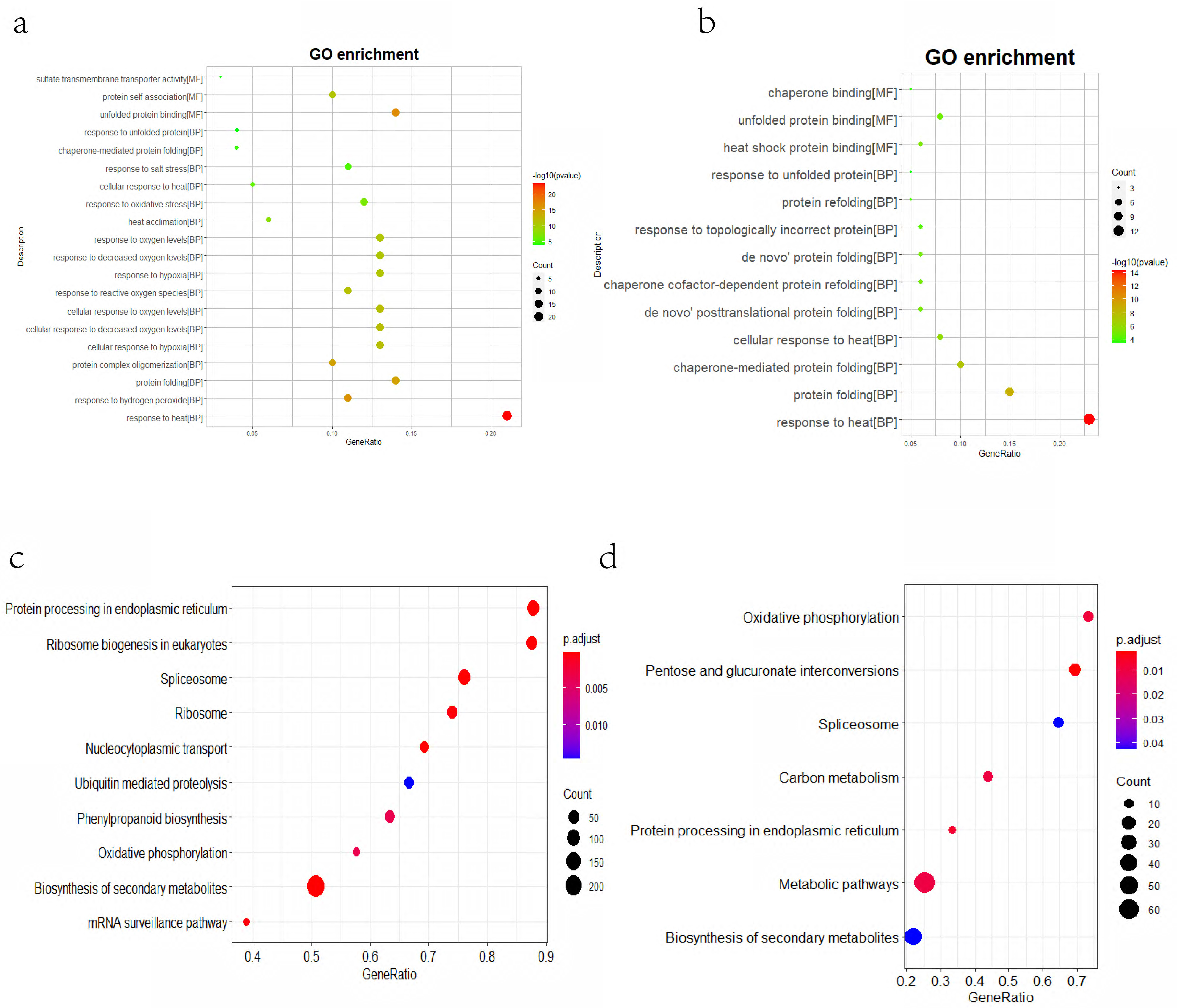

Supplement: Supplementary file 1 [file ijms-24-11081-s001.zip › sfigure1.jpg]

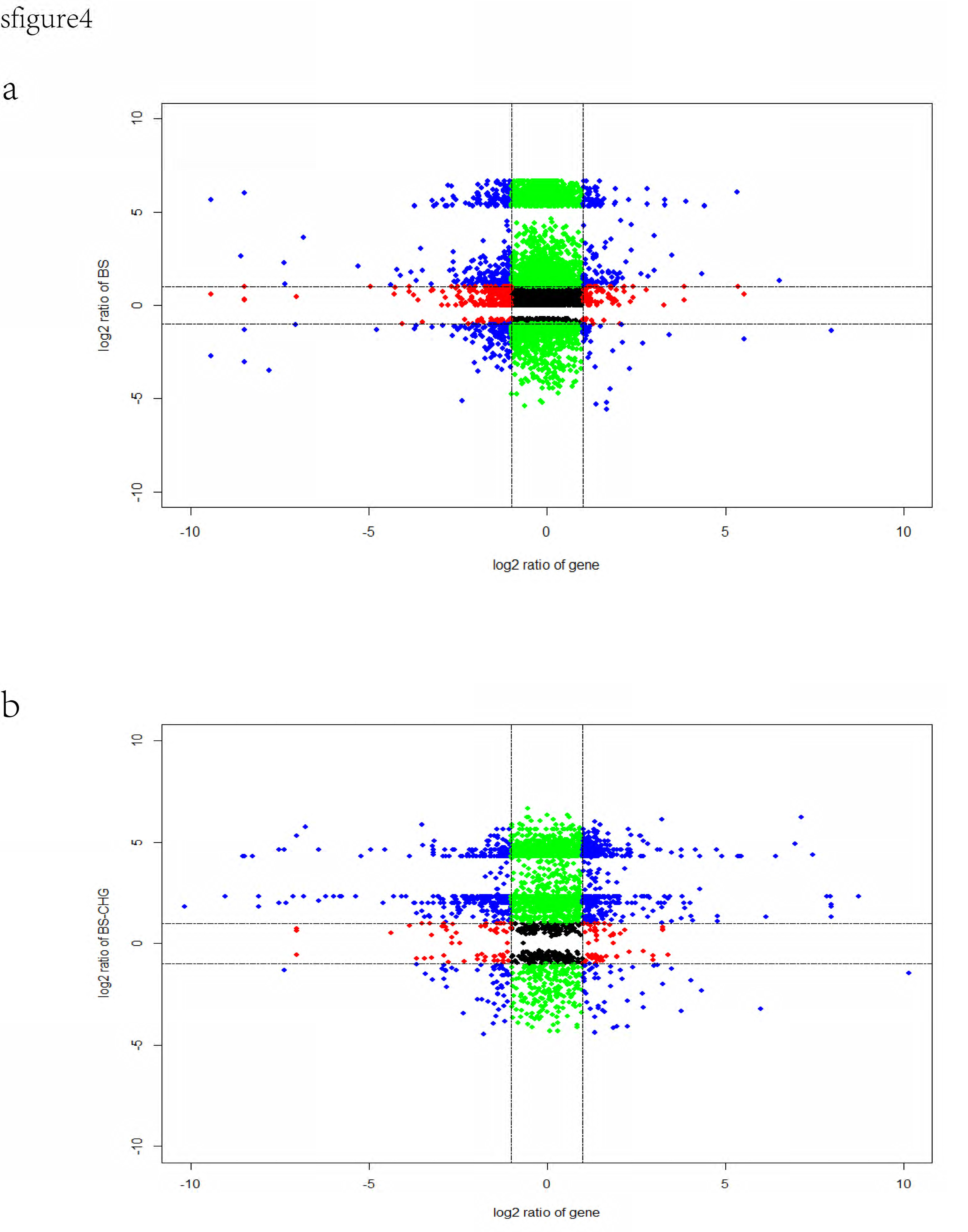

Supplement: Supplementary file 1 [file ijms-24-11081-s001.zip › sfigure4.jpg]
